# Swiss-CHAT: Citizens Discuss Priorities for Swiss Health Insurance Coverage

**DOI:** 10.15171/ijhpm.2018.15

**Published:** 2018-03-06

**Authors:** Samia A. Hurst, Mélinée Schindler, Susan D. Goold, Marion Danis

**Affiliations:** ^1^Institute for Ethics, History, and the Humanities, Geneva University Medical School, Geneva, Switzerland.; ^2^Department of General Internal Medicine, University of Michigan Medical Center, Ann Arbor, MI, USA.; ^3^Department of Bioethics, National Institutes of Health, Bethesda, MD, USA.

**Keywords:** Resource Allocation, Priority Setting, Public Participation, Universal Insurance System

## Abstract

**Background:** As universal health coverage becomes the norm in many countries, it is important to determine public priorities regarding benefits to include in health insurance coverage. We report results of participation in a decision exercise among residents of Switzerland, a high-income country with a long history of universal health insurance and deliberative democracy.

**Methods:** We adapted the Choosing Healthplans All Together (CHAT) tool, an exercise developed to transform complex healthcare allocation decisions into easily understandable choices, for use in Switzerland. We conducted CHAT exercises in twelve Swiss cities with recruitment from a range of socio-economic backgrounds, taking into account differences in language and culture.

**Results:** Compared to existing coverage, a majority of 175 participants accepted greater general practice gatekeeping (94%), exclusion of invasive life-sustaining measures in dying patients (80%), longer waiting times for non-urgent episodic care (78%), greater adherence to cost-effectiveness guidelines in chronic care (66%), and lower premium subsidies (51%). Most initially chose greater coverage for dental care (59%), quality of life (57%), and long-term care (90%). During group deliberations, participants increased coverage for out-of-pocket costs (58%) and mental health to current levels (41%) and beyond current levels for rehabilitation (50%), and decreased coverage for quality of life to current levels (74%). Following group deliberation, they tended to change their views back to below current coverage for help with out-of-pocket costs, and back to current levels for rehabilitation. Most participants accepted the plan as appropriate and fair. A significant number would have added nothing.

**Conclusion:** Swiss participants who have engaged in a priority setting exercise accept complex resource allocation trade-offs in healthcare coverage. Moreover, in the context of a well-funded healthcare system with universal coverage centered on individual choice, at least some of our participants believed a fully sufficient threshold of health insurance coverage was achieved.

## Introduction

Allocation decisions pose a challenge for health systems in all countries and even well established theories of distributive justice are not easily translated into practice when health-related allocation decisions are required. Procedural approaches have been proposed^1^ and public participation advocated^2^ to attempt to improve the fairness and legitimacy of priority setting. Determining fair priorities for healthcare coverage given limited resources has been the object of increasing discussion in the medical, bioethical, and philosophical literature.^3,4^ Ethical approaches may also compete with each other. For example, utilitarian approaches may prioritize the most cost-effective interventions, which can conflict with an approach that prioritizes fair equality of opportunity. A number of ethicists, most prominently Norman Daniels^1^ and Leonard Fleck,^5^ propose procedural approaches to priority setting, sometimes with public inclusion. Decisions about priorities in health coverage, however, often present medical and scientific complexity as well as competing needs and values. Making this complexity easily understandable and accessible to public deliberation remains a challenge.

We conducted a study to explore the priorities of a sample of the public in Switzerland, a country with a strong tradition of direct democracy, universal coverage, and high healthcare costs.^6^ Switzerland’s health system spent 9674 int$ (6468$ purchasing power parity) per capita on health in 2014 (source WHO; http://apps.who.int/nha/database/Key_Indicators/Index/en). The system is based on an individual mandate for insurance covering a federally defined basic package. Basic insurance is provided by dozens of private health insurance funds, with the 10 largest insurers covering over 80% of policyholders. Coverage for services included in insurance packages is 90% of the cost above the deductible (300 to 2500 CHF), with co-pays capped at 700 CHF per year for adults, and 350 CHF for children. The country’s 26 Cantons are responsible for the provision of care, The Confederation thus guarantees a health system where everyone must be affiliated with the level of basic health insurance. Premiums vary with the canton of residence, but cannot be risk-adjusted in other ways. Swiss citizens are involved to an unusual degree in policy setting for health, since they often have the opportunity to vote about healthcare issues. Yet the questions put to vote are rarely the sort of choices most relevant to questions of priority setting for resource allocation in healthcare. Most frequently, the public votes on *whether or not* they approve of one particular form of health coverage, and never on whether they *would prioritize one kind of intervention or another.* Even the more comprehensive surveys previously conducted in Switzerland, such as the Bertelsmann Stiftung’s *Gesundheitsmonitor,* ask about opinions regarding funding on individual sectors of healthcare. Public input has been largely about abstract, macro-level questions, and not about what trade-offs members of the public would be willing – or unwilling – to make. Unless choices are posed as trade-offs within a constrained budget, it is difficult to translate public priorities into health policy.

In this paper, we report the trade-offs members of the Swiss public might be willing or unwilling to make in their health insurance coverage. Qualitative results on arguments and principles underlying participants’ choices will be published elsewhere.^7^

## Methods

### Adapting the Choosing Healthplans All Together Tool

We adapted the Choosing Healthplans All Together (CHAT; usechat.org, © University of Michigan) exercise that has been previously developed for similar exercises in the United States, New Zealand, and India.^8-10^ Initial development of this exercise has been described elsewhere.^11^ In contrast to other collective decision tools, such as ethical matrix analysis,^12^ the CHAT tool allows trade-offs between different possible areas of healthcare – rather than the analysis of a single technology. It also integrates technical knowledge in the structure of the game while letting participants input their own values, thus enabling trade-offs between technically complex options to be made by members of the public. Past research indicates that lay members of the public, including the disadvantaged, find these simulations exercises enjoyable, understandable, and informative, and consider the group process and decision fair. This research demonstrates public-spirited decisions and reasoning,^13^ changing attitudes toward rationing^11^ and changing (arguably more prudent) individual priorities after participation in CHAT and the similarly structured REACH exercise.^11,14,15^ Adaptation of the CHAT tool to Switzerland required: (1) identification of the most relevant questions, (2) revising the materials used in the exercise, (3) development of scenarios fitting the Swiss healthcare system, and (4) translation into local languages. To identify relevant questions, we held preparatory discussions with Swiss “key informants”: physicians, members of parliament from different political parties, and patient representatives involved in issues regarding the healthcare system. Our intent was to complement our understanding of what the most relevant questions were. Based on these discussions, we decided to focus on coverage decisions regarding various health conditions (chronic, acute, maternity, etc), rather than different types of health services (pharmacy, tests, specialty, etc) and also included options designed to assess attitudes regarding aspects of healthcare financing such as the level of co-pay or premium subsidies. To design trade-offs based on realistic scenarios, we worked with Milliman, an international actuarial company familiar with the Swiss healthcare system and experienced with adaptations of the CHAT project for different US states, to create insurance benefit options that would be compatible with the Swiss healthcare system and relevant there (see Box 1 and Supplementary file 1 for benefit descriptions; lower numbered tiers are less extensive and expensive). We then created scenarios – or health events – to help participants think about and appreciate the practical consequences of their benefit choices. Finally, we translated the material into German, French, and Italian. Translations were back translated and checked by individuals familiar with these languages.

**Box 1. Domain Descriptions**

**Optional Categories**

1. **Severe injury or illness care:** Care for sudden, bad injury or illness. Examples – sudden liver failure from food poisoning; massive injuries from an accident; a very premature and sick newborn.

2.** Complicated Chronic Illness:** Care of serious long illnesses like diabetes, heart failure, rheumatoid arthritis. These illnesses are complex and need lots of medical care to keep patients functioning as much as possible.

3. **Dental:** For care by dentists to prevent and treat dental problems. (Surgery of the jaw after injury, for example, is not here but under severe injury).

4. **Vision: **Testing and correcting for problems with eyesight that can be corrected with glasses or contact lens. Does not include other eye care. Laser treatment of the retina for diabetics would be covered by complex chronic illness.

5. **End-of-life care:** For patients with a terminal illness who are likely to die in a few months.

6. **Episodic care:** Treatment such as office visits, tests, and drugs for short term problems, such as a sore knee, constipation, cough, heart burn, or skin rash, but also short-term urgent problems like appendicitis.

7. **Chronic illness care:** Routine checkups and care of chronic conditions that are new and not complicated.

8. **Sexual and reproductive care:** for care of birth control, pregnancy, sexual function, and fertility.

9. **Mental and behavioral care:** For detecting and treating mental illness. May also cover Behavioral Health problems such as drug and alcohol abuse.

10. **Quality of Life:** For problems that are not badly disabling but affect quality of life, such as injuries affecting athletic performance. These problems affect a person’s ability to act, look, or feel well.

11. **Prevention:** To help prevent many diseases or illnesses. To identify medical problems as early as possible. There are no copays for preventive services.

12. **Rehabilitation:** To restore or improve ability to do daily activities. This includes walking, speaking, bathing, eating and critical work functions. Often needed if a person has a stroke, a joint replaced, or a limb removed.

13. **Long term care:** To pay for the care of a person who can no longer function independently that is provided at home or an institutional setting.

**Required Categories **

14. **Out of pocket costs and Premium:** This is the money that individuals pay to use health care services. Co-payments are not required for basic preventive services or routine screening tests.

15. **Premium subsidy:** Subsidies given to lower income persons and families.

16. **Specialists:** This is access to specialists and the range of choice of doctors and hospitals.

17. **Time with the doctor:** This is the frequency and length of medical visits.

### Participants

In collaboration with TA-Swiss, we defined the number of focus groups required for a sampling of the Swiss population that would illustrate the diversity of organizational and cultural aspects within our healthcare system. Participants were recruited throughout Switzerland through a market research agency (Yxplora, Zürich). Participants were eligible if they were residents of Switzerland aged over 18 years and had consented to participation. We excluded candidates with insufficient knowledge of the local language (either French, German or Italian). Volunteering participants were selected based on five stratification criteria: rural or urban, gender, age, socio-economic level and language. We did not recruit according to health status given the private nature of that information. In order to recruit as broadly as possible geographically, we conducted focus groups in four French-speaking cities (Geneva, Lausanne, Bienne, Sion), six German-speaking ones (Bern, Basel, Zurich, St-Gallen, Chur, Luzern), and two Italian-speaking ones (Lugano and Bellinzona), recruiting each time to include participants from both rural and urban areas. This allowed us to recruit from the entire country.

### The Exercise

The CHAT exercise brings together small groups of people for approximately three hours. It confronts people through a simulation exercise with the problem of prioritizing benefits to be covered by basic health insurance Participants use a pie-shaped board on which the various benefit options are arrayed to make their choices. The board is shown in Figure and the benefit options outlined in Box 1. We deliberately designed the board so that no single level (basic, medium, or high) would represent the current Swiss coverage in every area. To make choices, participants are given 50 stickers representing units of currency, for use in the selection of their benefit packages. Each sticker represents 1/50th of the average annual cost of health coverage for one person. Each group participated in four rounds of decision-making: alone, in groups of three, in full moderated deliberation with the entire group, then alone again using what they had learned in the previous cycles. Participants were guided in all rounds to first choose benefits at the basic level before selecting higher coverage levels. A CHAT manual written in simple French, German or Italian that described the benefits and the number of markers required to cover them was also given to participants.

**Figure:**
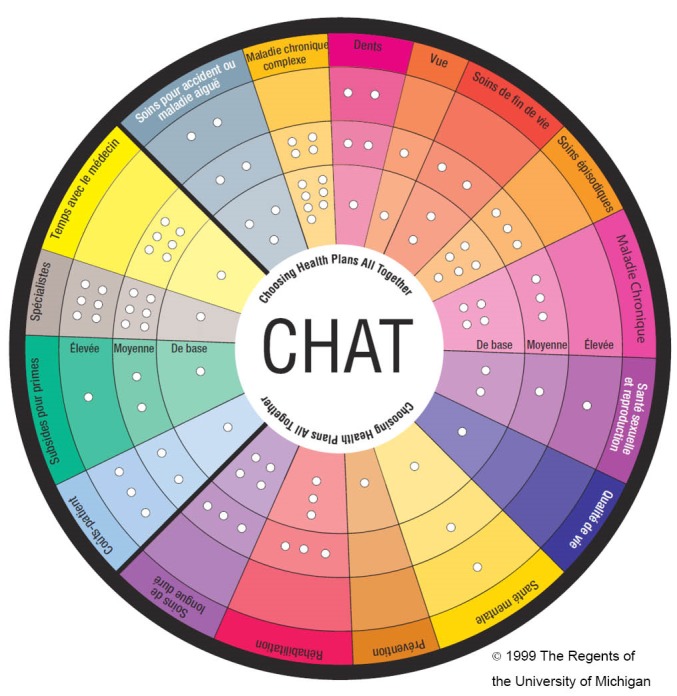
The Swiss-CHAT Board.

### Data Collection 

During each round of the exercise, participants’ choices for insurance benefits were recorded. The location of stickers on their individual CHAT boards serves as a recording of the participants’ priorities at various stages in the exercise. Before and after participation in the CHAT exercise, participants completed a survey (pre and post exercise surveys) to determine socio-demographic characteristics and attitudes towards heath care and any change of opinions following the completion of the exercise. All materials were available in French, German and Italian.

### Data Analysis 

Participants’ socio-demographic characteristics and their insurance choices were analyzed using descriptive statistics. We conducted bivariate analysis to examine socio-demographic characteristics and regional location associated with insurance choices. Pre- and post-exercise surveys were analyzed using appropriate non-parametric statistical tests.

###  Protection of Human Participants 

Each participant was contacted and informed about the study, and gave informed consent. Personal participant data were kept strictly confidential. Participants were paid 75 Swiss francs for their travel expenses and participation.

## Results

### Participant Characteristics

Volunteers (N = 175) participated in 12 groups of 14-16 individuals each. Participant characteristics are shown in Table 1. Most (57%) rated their own insurance coverage as excellent or very good, 30% as acceptable, and 10% as skimpy. In reporting their real-life health insurance choices of paying either higher premiums or higher deductible, they reported different strategies with 35% opting for a low premium with high deductibles, 32% for a mid-range premium and deductible, and 30% for a high premium but low deductibles. Despite being covered in a universal coverage health system, 20% of participants reported forgoing medical care for reasons of cost in the past 12 months.

**Table 1:** Participant Characteristics

Variable		
Age	Median	44
	Range	18-88
Gender	Male	46%
	Female	54%
Language	French	33%
	German	50%
	Italian	17%
Nationality	Swiss	80%
	Double	5%
	European	10%
	Other	5%
Marital status	Married	39%
	Single	25%
	Partnered	15%
	Divorced	20%
	Widowed	1%
School level	Primary	3%
	Apprentice	38%
	Secondary	9%
	University	36%
	Other	14%
Monthly income	Median	5000-6999 CHF
	Minimum	None
	Maximum	>15 000 CHF
Self-reported health	Excellent/VG	41%
	Good	33%
	Fair/Poor	27%
Health insurance strategy	Low premium – high deductible	35%
	Middle premium and deductible	32%
	High premium – low deductible	30%

###  Attitudes Towards the Healthcare System

Before the exercise, most participants (55%) assessed their knowledge of the health system as average (Table 2). Most (80%) reported that the main reason for insurance was to pay for daily costs of care as opposed to paying for catastrophic costs. A little under half (44%) thought that protection should be the same for everyone, with the same proportion disagreeing that it was reasonable to limit what is covered by health insurance given the rising cost of healthcare. When prompted about their concerns regarding the future of the health system (Table 3), half agreed that “the amount I have to pay for healthcare will be more than I can afford” (50%) and that “The types of services that are covered by insurance will be reduced (51%). Although few were concerned that they would personally become excluded from the health system (5%), more were concerned that “care will no longer be offered to all” (27%), that “the quality of healthcare will be reduced” (35%), that “Healthcare rationing will be put into place” (19%), “Waiting times for treatment will increase” (21% and especially that “Choice (of doctors, hospitals, or health plans) will be reduced” (41%). One fifth (21%) was concerned that “Healthcare costs will drain resources from other societal needs.”

**Table 2:** Participant Attitudes

		**Before**	**After**
Knowledge of the health system	Excellent/Very good	31%	61%
	Average	55%	36%
	Low	14%	3%
Reason for insurance	Pay daily	80%	30%
	Against catastrophic	20%	70%
Equal protection	Same	44%	51%
	Same for essential	40%	33%
	Not same	17%	15%
Reasonable to limit	Agree	18%	28%
	Moderate	37%	25%
	Disagree	44%	45%
Government should limit drug prices	Agree		73%
	Neutral		18%
	Disagree		7%
Opinion of designed plan	Appropriate for basic coverage	65%
	Too low for basic coverage	26%
	Too rich for basic coverage	5%
Fairness of designed plan	Plan is fair for ill persons		78%
	Uncertain		19%
	Plan is unfair for ill persons		1%
What would you have spent additional markers on?	Nothing		38
	Dental		28
	Specialists		28
	Vision		20
	Rehabilitation		19
	Accidents and acute care		17
	Chronic disease		16
	Complex chronic disease		14
	Long-term care		14
	Premium subsidies		12
	Time with the doctor		11
	Out-of-pocket costs		10
	Prevention		8
	Mental health		7
	End of life		5
	Episodic		5
	Sexual and reproductive health		5
	Quality of life		4
	Everything		2

**Table 3:** Participant Concerns

	**Before**
The amount I have to pay for healthcare will be more than I can afford	50%
Choice (of doctors, hospitals or health plans) will be reduced	41%
The types of services that are covered by insurance will be reduced	51%
The quality of healthcare will be reduced	35%
Care will no longer be offered to all	27%
Waiting times for treatment will increase	21%
Healthcare costs will drain resources from other societal needs	21%
Health care rationing will be put into place	18%
My own exclusion from health system	5%
Nothing concerns me about the rising cost of health insurance	5%

###  Participant Priorities

Participant priorities in the first, third, and fourth round are outlined in Table 4. Compared to current coverage, a majority of participants accepted greater general practice gatekeeping for access to specialists (94%), exclusion of invasive life-sustaining measures in dying patients (80%), longer waiting times for non-urgent episodic care (78%), greater adherence to cost-effectiveness guidelines in chronic care (66%), and lower premium subsidies (51%), and lower than current levels for mental health (45%). Most chose greater coverage for long-term care (90%), dental care (59%), and quality of life (57%).

**Table 4:** Coverage Options

	**Respondents (N = 175) **				
		** No coverage **	** Level 1 **	** Level 2 **	**Level 3 **
	** Rounds **					
Dental	1	5%	35%	43%	16%
	3	-	43%	57%	-
	4	7%	39%	44%	
Vision	1	11%	42%	46%	
	3	9%	75%	17%	
	4	11%	32%	58%	
End of life*	1	10%	70%	19%	
	3	-	82%	18%	
	4	4%	73%	23%	
Episodic	1	18%	60%	22%	
	3	-	91%	9%	
	4	13%	78%	9%	
Chronic disease	1	6%	60%	33%	
	3	-	66%	34%	
	4	-	66%	33%	
Sexual and reproductive*	1	17%	54%	21%	9%
	3	-	91%	9%	-
	4	10%	69%	15%	6%
Quality of life	1	42%	57%		
	3	74%	26%		
	4	54%			
Mental health	1	5%	40%	33%	23%
	3	-	26%	41%	33%
	4	2%	30%	42%	26%
Prevention*	1	12%	87%		
	3	-	100%		
	4	8%	91%		
Rehabilitation	1	5%	60%	35%	
	3	-	50%	50%	
	4	2%	63%	35%	
Long-term care	1	11%	57%	33%	
	3	-	92%	8%	
	4	4%	69%	27%	
Out-of-pocket costs	1		45%	39%	16%
	3		42%	58%	-
	4		53%	42%	6%
Premium subsidies	1		51%	33%	16%
	3		49%	25%	26%
	4		51%	30%	18%
Access to specialists	1		61%	33%	6%
	3		83%	17%	-
	4		80%	19%	1%
Time with the doctor	1		87%	13%	
	3		100%	-	
	4		93%	5%	
Accidents and acute care*	1	3%	40%	43%	14%
	3	-	42%	58%	-
	4	1%	36%	55%	8%
Complex chronic disease	1	23%	58%	18%	
	3	-	76%	24%	
	4	7%	67%	26%	
Current coverage					
Unavailable option					

Percentages are valid percent. * Difference between language regions in round 1: *P* ≤ .01.

We found regional differences in participants’ initial choices for coverage of end of life care, sexual and reproductive health, prevention, and accidents and acute care. Italian speakers chose no coverage or second-tier coverage for end of life care more frequently than participants in other language regions who mostly chose first-tier. They also chose no coverage or third-tier coverage for sexual and reproductive health more frequently than other participants, who mostly chose first-tier coverage. They chose third-tier coverage for accidents and acute care, with German – and French speakers mostly covering either first – or second-tier in this category. German speakers mostly chose not to cover prevention, whereas others did.

During group deliberations, participants tended to change their views to increase assistance with out-of-pocket costs (58%) and coverage for mental health to current levels (41%), to increase rehabilitation beyond current levels (51%), and to decrease coverage for quality of life to current levels (74%). Following group deliberation, participants responded to the fourth round on their own and tended to change their views back to below current coverage for help with out-of-pocket costs, and back to current levels for rehabilitation. A majority remained in favor of the current level of coverage for mental health (42%) and quality of life (54%).

When asked what they would have spent additional markers on, participants chose dental care, access to specialists, and vision care most frequently. The most frequent answer, however, was “nothing.”

### Post-exercise Attitudes

Most participants (65%) assessed their final coverage plan as appropriate for basic coverage, and reported that they found it fair for ill persons (78%) (Table 2). Following the exercise, 61% of participants reported their own knowledge of the health system as excellent/good. Most (70%) now reported that the main reason for insurance was to pay for catastrophic costs as opposed to paying for daily costs of care (*P* < .00). More participants agreed that benefits should be the same for everyone, with fewer reporting that this should only apply to the “really essential benefits” (*P* < .00). A similar number as before (45%) disagreed that it was reasonable to limit what is covered by health insurance given the rising cost of healthcare. However, 73% agreed that “government should establish reasonable price limits on the cost of new drugs, even though some say this might limit medical innovation.”

**Table 5:** Cost-Control Strategies Preferred by Participants

**Which actions do you think would be MOST helpful to control the cost of healthcare in this country?**	
Promote the use of generic drugs	46%
Put price controls on the cost of new drugs	42%
Reduce over-use of treatment that accomplishes very little	35%
Have a government-financed program for everyone	30%
Require patients to pay more if they do not follow medical advice that would keep them healthy	15%
Have stricter standards for the use of expensive new medical technology	14%
Use “managed care” than will establish control of health costs	12%
Make choices about the direction of treatment	11%
Reduce payments to hospitals, doctors and other health providers	10%
Have a cantonal-financed program for a part of the population	10%
Remove supplementary insurance	9%
Have consumers pay more if they choose more expensive treatment options	8%
I would do nothing. I do not think that higher costs are a problem	2%
Nothing will be helpful to control the cost of healthcare in this country	1%

When asked about which cost-control mechanisms they thought would be most helpful, participants ranked the use of generic drugs highest (46%) (Table 5), followed by price controls on the cost of new drugs (42%) and reduction of over-use of treatment that accomplishes very little (35%). No mechanism was deemed the most helpful by a majority of participants.

When asked about which cost-control mechanisms they though were most acceptable (Table 6), participants ranked restriction of coverage for treatment that is not critical for basic functioning and long life highest (49%), followed by restriction of coverage for treatment that does meet national standards for effectiveness (43%) and higher standards for when expensive new technology can be used (37%). Here also, no mechanism was deemed the most acceptable by a majority of participants. Most participants (55%) did however find an increase in premiums to be the least acceptable cost-control mechanism.

**Table 6:** Cost-Control Mechanisms

	**MOST Acceptable**	**LEAST Acceptable**
Restrict coverage of treatment that is not critical for basic functioning and long life	49%	14%
Restrict coverage of treatment that does not meet national standards for effectiveness	43%	15%
Have higher standards for when expensive new technology can be used	37%	16%
Limit the network of doctors and hospitals that can be used	32%	28%
Except for emergencies, have longer waiting times for services	20%	27%
Increase co-payments that individuals pay for services	11%	41%
Increase the amount of the premium paid by consumers	6%	55%

## Discussion

As tensions arise between the pressures to control healthcare expenditures and to accommodate high public expectations about healthcare, trade-offs within health systems can be politically fraught. Knowledge of public attitudes towards such trade-offs is often lacking. Our study showed that, when given an opportunity to convene and discuss coverage decisions, members of the public from a wealthy healthcare system with universal coverage were able agree and make trade-offs regarding resource allocation in their health insurance coverage. Participants accepted greater general practice gatekeeping, longer waiting times for episodic care, greater adherence to cost-effectiveness guidelines in chronic care, lower premium subsidies, and less access to invasive care at the end of life. Instead, they prioritized increased coverage for long-term and dental care. Most participants assessed the final coverage plan as appropriate and fair, with a substantial proportion declining the chance to allocate additional funds to anything. No cost-control mechanism was accepted by a majority of participants. However, generic substitution and controls on drug prices as well as controls on the use of treatments with little utility were deemed most helpful, while limits on coverage for interventions that are not critical or do not meet standards of effectiveness were deemed most acceptable. Participants changed some of their views during the exercise, with more reporting that the main reason for insurance was to pay for catastrophic costs, and that benefits should be the same for everyone. Participants accepted the plan as appropriate and fair, and a significant number would have added nothing. This is of particular interest. Debates on resource allocation in health systems often presume that needs are endless and that beneficiaries would, when asked, always opt to include everything. Our findings suggest that, in the context of a well-funded healthcare system with universal coverage centered on individual choice, at least some participants believe a threshold of sufficiency has been reached.

What participants chose not to fund is of particular interest. Our qualitative results showed that their decision to limit access to invasive treatments at the end of life was cast as better medicine rather than a trade-off. Here too, at least some of our participants seemed to reach a saturation of their healthcare needs.^7^ This finding is also in line with Swiss end of life studies, which show a progression of limiting invasive care at the end of life.^16^ This seems to stand in contrast with NICE guidelines which aim to give “special consideration” to life extending interventions at the end of life, for example with the goal of providing more time to plan for death,^17^ but does align with the notion that preventing death should not be the main priority in end-of-life care.^18^ Since it is an example of Swiss participants actively wanting less, it also suggests that policies such as *Choosing Wisely,* which were recently implemented in Switzerland (see http://www.smartermedicine.ch), could meet with more public approval than might be thought.

Participants also assigned lower priority to interventions which lacked proof of effectiveness or showed benefits that were too small, mostly to increase coverage for long-term care. In doing so, they departed from more usual public discussions on resource allocation on two points. First, these discussions tend to center on whether we should limit interventions that are simultaneously very effective and very costly. Although disinvestment of interventions with little added value or proof - regardless of their cost - is often mentioned, it is rarely practiced.^19^ Second, public discussions tend to leave aside the possibility that medical care could be traded-off in favor of benefits viewed as being outside the scope of medicine. Here, participants were willing to give up some medicine in order to alleviate burdens in old age. This finding is similar to willingness to trade-off health benefits for other forms of social support that have been found in other CHAT and REACH exercises.^20-22^

Although individual choice is central in the Swiss health system,^6^ our participants accepted greater gatekeeping than is included in basic coverage in Switzerland. This is in line with the popularity of insurance contracts offering greater gatekeeping in exchange for lower premiums. In a country where the population has repeatedly voted to maintain greater consumer choice even at a higher cost, however, this remains a striking observation. It may, of course, stem from our recruitment strategy, which prioritized equal rather than proportional representation of different social groups. Intriguingly, however, it may also suggest that deliberation with information input in real time yields results different from those of a popular vote.^23^ It should be noted that our findings did not contradict the importance of consumer choice to Swiss citizens as such: more direct access to specialists was high on the list of what our participants would have funded if given additional means.

Differences between regions were also intriguing in this respect. Universal basic coverage is decided centrally in Switzerland and is identical in all regions. It is plausible that greater public input would lead to different trade-offs across the language regions or Cantons.

Our study has several limitations. Most obviously, this was a hypothetical exercise. Participants, however, remarked that results could be brought to bear on real decisions in the Swiss context and this could have decreased the difference between choices made in this exercise and choices they would have made in practice. Although the duration of the exercise was insufficient for a full deliberation of all implications, it did allow participants to focus on the points they found to be most important. After discussion, participants often found the level of coverage that is currently available in practice to be their preferred option. It is possible that knowledge of existing coverage policy could have exercised a norming effect. We limited this effect by not identifying the current level of coverage during the exercise, and by designing the board in such a way that it could not be guessed from its structure. The participants in this research are not a representative sample in the statistical sense. Participation in focus groups involves some matters of convenience – we include individuals who are available at the time and place that the groups are being convened. Within that constraint, the recruitment firm sought individuals who varied in socio-economic background, as shown in Table 1 which reports respondent characteristics. The frequency of their responses cannot be interpreted as representing the prevalence of these views within the Swiss population. Instead, our sample represents the diversity of profiles of people living in Switzerland. Use of an agency that usually recruits participants for market research may have meant that our participants were over selected for their interest in market research. However recruitment intentionally included diverse socio-economic profiles and our demographic data suggests that this was successful. While we were not able to select on the basis of health status or experiences of illness, our sample did include people with a range of health states. Since the views of both the sick and healthy are relevant to decisions regarding healthcare coverage, homogenous groups of either sick or healthy participants would have weakened our groups’ ability to recognize some important tradeoffs. Despite lack of stratification for health-status, we did obtain both sick and healthy participants in our groups. We were unable to include participants with insufficient use of local languages to allow deliberation. In Switzerland, 3% of the total population do not speak any of the national languages^24^; we thus excluded a group that, while small, is particularly vulnerable. We paid participants and this could have led to an overrepresentation of participants in need of the money. For this reason, recruitment was targeted to obtain a diversity of economic levels and here too our demographic data indicates that this was successful. As is usual with this type of recruitment, any generalizations to different cultural or structural contexts must be cautious. We used a highly structured focus group methodology and this may have influenced some of the responses. This was necessary, on the other hand, to explore specific trade-offs as this required that participants focus on these choices rather than providing us with only more general views of how priorities ought to be set in the health system.

## Conclusion

Public deliberation about healthcare priorities in a country with a long tradition of democracy and solidarity seems to yield trade-offs different from current coverage and from strictly personal interest of participants. Participants were able to engage with complex resource allocation trade-offs in healthcare coverage and to agree on a set of priorities. They tended to disinvest interventions with little added value or proof – regardless of their cost- mostly to increase coverage for long-term care. We also found that, in the context of a well-funded healthcare system with universal coverage centered on individual choice, at least some of our participants were inclined to believe that a sufficient level of health insurance coverage can be achieved.

Grey cells stand for current coverage and black ones for unavailable options. Bold figures are the option chosen by the greatest number of participants.

## Acknowledgments

The authors would like to thank Milliman for their attentive actuarial work, Charlotte Ghisler and Paola Santini for outstanding moderation of the focus groups, as well as key informants and group participants for shaping this project and making it possible.

This work was funded by the National Institutes of Health Department of Bioethics, USA, Käthe Zingg Schwichtenberg fund at the Swiss Academy of Medical Sciences, Switzerland, the Institute for Ethics, History, and the Humanities at the Geneva University Medical School, Switzerland, and the Swiss National Science Foundation, Switzerland (grant PP00P3_123340).

## Ethical issues

This study was designated exempt from ethics review by the chair of the Geneva research ethics commission and the Office of Human Subjects Research Protection at the National Institutes of Health.

## Competing interests

Dr. Goold and Dr. Danis, and their institutions, may benefit from paid licenses for the use of CHAT©.

## Authors’ contributions

SAH, SDG, and MD contributed to the conception and design of the study, SAH and MS collaborated in the acquisition of data, all authors contributed to the analysis and interpretation of data, SAH and MD obtained funding, adminitrative and material support, performed statistical analysis and supervised the study. SAH wrote the first draft. All authors provided critical revisions of the manuscript for important intellectual content and approved the submitted paper.

## Authors’ affiliations

^1^Institute for Ethics, History, and the Humanities, Geneva University Medical School, Geneva, Switzerland. ^2^Department of General Internal Medicine, University of Michigan Medical Center, Ann Arbor, MI, USA. ^3^Department of Bioethics, National Institutes of Health, Bethesda, MD, USA.

## Disclaimer

The views reported here are those of the authors and do not necessarily reflect the policies of the National Institutes of Health or the US Department of Health and Human Services.

## Supplementary Files

Supplementary file 1. Benefit Options for the Swiss-CHAT Exercise.

## Key messages

**Implications for policy makers**

- Given appropriate tools, public participation in complex trade-offs regarding priority setting in healthcare is possible.
- Participants assigned lower priority to interventions which lacked proof of effectiveness or showed benefits that were too small, mostly to increase coverage for long-term care.
- In the context of a well-funded healthcare system, at least some participants believe that a sufficient threshold of health insurance can be fully achieved.

**Implications for the public**

In distributing healthcare resources, it is important to compare interventions to each other in order decide which ones to cover: public participation in such trade-offs is important and possible. In this study of Swiss citizens, the decisions reached by participants differed from the current distribution in the Swiss healthcare system. Participants in this study did not agree about all choices, but ultimately most of them found the plan they had developed together to be a fair one.
