# Supplementary material for: Swiss-CHAT: Citizens Discuss Priorities for Swiss Health Insurance Coverage
Source: Int J Health Policy Manag. 2018 Mar 6;7(8):746–54. doi: 10.15171/ijhpm.2018.15 (PMC6077280; doi:10.15171/ijhpm.2018.15)
Supplement: Supplementary file 1 — Benefit Options for the Swiss-CHAT Exercise. [file ijhpm-7-746-s001.pdf]

Supplementary file 1. Benefit Options for the Swiss-CHAT Exercise

Optional Categories:

1. Severe injury or illness care: Care for sudden, bad injury or illness. Examples – sudden liver failure from food poisoning; massive injuries from an accident; a very premature and sick newborn.

> Tier 1: All emergency care is covered to try to save the person's life. Treatments are those proven to work. If these treatments do not work or no such treatment exists, supportive care is covered.
>
> Tier 2: All emergency care is covered to try to save the person's life and establish basic functioning. Perhaps there are no proven treatments for a condition. Perhaps well-proven treatments have not worked for an individual. Then insurance will cover treatments that have some proof that they might work.
>
> Tier 3: Includes Tier 2. For individuals at the end stage of their disease, this also covers very costly care that has a very small chance of helping or extending their life.

2. Complicated Chronic Illness: Care of serious long illnesses like diabetes, heart failure, rheumatoid arthritis. These illnesses are complex and need lots of medical care to keep patients functioning as much as possible.

> Tier 1: Doctor must follow guidelines for the least costly ways to manage complex chronic illness. Though the covered tests, treatments and drugs are effective for most people, they may not work quite as well as more costly option.
>
> Tier 2: Includes Tier 1. Also covers the more costly options, including extensive treatments that usually improve patients' health or function. For instance, knee replacement if arthritis makes walking impossible.

3. Dental: For care by dentists to prevent and treat dental problems. (Surgery of the jaw after injury, for example, is not here but under severe injury).

> Tier 1: Dental care is covered under health insurance if your doctor states that the dental problems are caused by your medical condition.

Tier 2: Cleanings and x-rays are covered once a year without co-payment. Basic dental services are 80% covered, such as emergencies, cavities, oral surgery. Pays 50% of crowns and bridges. Maximum coverage is 1,000 CHF/y.

Tier 3: Crowns and bridges are covered. Maximum is 3000 CHF/y.

4. Vision: Testing and correcting for problems with eyesight that can be corrected with glasses or contact lens. Does not include other eye care. For instance, laser treatment of the retina for diabetics would be covered by complex chronic illness.

Tier 1: Covers Vision Care, which includes vision testing once a year, if needed. Covers 100 CHF towards glasses every 5 years, but not contact lenses.

Tier 2: Covers 180 CHF towards glasses or contact lenses every 5 years.

5. End-of-life care: For patients with a terminal illness who are likely to die in a few months.

Tier 1: Palliative care is covered to treat pain and other symptoms. This also covers emotional and spiritual support. Restricted hours and one visit a day in home care. Palliative care in hospital is covered for care of more intense symptoms.

Tier 2: Palliative care is covered. More hours a day and equipment available at home. Palliative care in hospital is covered for care of more intense symptoms. Also, if the patient or family wants, care will be covered even if it can only help delay death for a few days, weeks or months. This could include hospital intensive care, cardiac resuscitation, and breathing machines.

6. Episodic care: Treatment such as office visits, tests, and drugs for short term problems. This includes problems such as a sore knee, constipation, cough, heart burn, or skin rash. This also covers short-term urgent problems like appendicitis.

Tier 1: All emergencies and urgent care are dealt with quickly. When the problem is not urgent, patients may wait a couple weeks or up to 2 months for medical appointments, tests or surgery. You have limited provider choice. Doctor is required to follow guidelines based on cost effectiveness.

Tier 2: All emergencies and urgent care are dealt with quickly. When the problem is not urgent, patients wait a couple weeks or less for appointments, tests or surgery. You have

wider provider choice. Doctors are not as restricted by guidelines based on cost effectiveness.

7. Chronic illness care: Routine checkups and care of chronic conditions that are new and not complicated. This is to maintain good health. These are conditions such as asthma, high blood pressure, diabetes.

Tier 1: Doctor is required to follow guidelines for the least costly ways to manage chronic illness. These guidelines suggest tests, treatments and drugs that are effective for most people. They may not work quite as well as more costly alternatives.

Tier 2: Doctor can order any tests, treatments and drugs that may help the patient. The doctor does not have to follow guidelines.

8. Sexual and reproductive care: for care of birth control, pregnancy, sexual function, and fertility.

Tier 1: Includes all services for pregnant women. Other sexual health and reproductive services are included only if the need is related to or could seriously complicate disease.

Tier 2: Low cost services (simple procedures, cheap ongoing or moderate-cost, short-term medicines) that aim to prevent or cause pregnancy, or restore sexual function whether or not due to disease.

Tier 3: High cost services that aim to prevent or lead to pregnancy, or restore sexual function.

9. Mental and behavioral care: For detecting and treating mental illness. May also cover Behavioral Health problems such as drug and alcohol abuse.

Tier 1: For severe mental health disorders covers inpatient and outpatient therapy and drugs. This includes illnesses such as bipolar disease, severe depression and anorexia. This does not cover Behavioral Health problems. Alcohol and drug abuse treated by the primary care doctor.

Tier 2: Besides Tier 1, covers less severe mental health problems, as well as behavioral health problems, with out-patient therapy and medications, and more intense outpatient follow-up for all mental health disorders. Alcohol and drug abuse is managed by

specialized clinics. Tier 2 also covers hospital in-patient drug and alcohol abuse treatment programs. Patients must meet medical criteria.

Tier 3: Besides Tier 2, also covers more intensive and accessible services for alcohol and drug abuse. Provides financial help for family members who care for a mentally disabled relative.

10. Quality of Life: For problems that are not badly disabling but affect quality of life. These problems affect a person's ability to act, look, or feel well. Examples: Injuries affecting athletic performance.

Tier 1: Treatments aimed at improving performance or well being are offered if patient expresses a strong preference. Examples are: Spa and massage therapy. Access to a sports trainer. Medical and surgical treatments needed to address cosmetic concerns.

11. Prevention: To help prevent many diseases or illnesses. To identify medical problems as early as possible. There are no co-pays for preventive services.

Tier 1: Covers wellness exams, screening tests and vaccines. Includes care to promote healthy behavior such as smoking cessation. Includes obesity treatment for people over a certain weight. Note that gastric bypass in case of obesity with already developing consequences would be covered under complex chronic. Interventions must meet national standards for being most effective. This includes such care as flu shots, PAP tests at certain ages, colon exam at age 50, cholesterol screening and others. Travel medicine advice is included.

12. Rehabilitation: To restore or improve ability to do daily activities. This includes walking, speaking, bathing, eating and critical work functions. Often needed if a person has a stroke, a joint replaced, or a limb removed.

Tier 1: Physical therapy in case of mild injury up to 10 sessions. This includes as much as 10 CHF/day for rehabilitative spa treatment up to 21 days/year. Covers all needed rehab services to improve basic functions. Covers artificial limbs but not patient equipment or supplies at home. Basic supplies and equipment are covered that are needed for daily activities like a manual wheelchair.

Tier 2: Covers 50% of the cost of expensive equipment, such as electric wheelchairs, more advanced artificial limbs. Lower threshold for assistive equipment.

13. Long term care:

Tier 1: 35% of the cost for care of a person who can no longer function independently that is provided at home or an institutional setting. Respite care for 2 weeks per year is 100% covered.

Tier 2: 60% of the cost for care of a person who can no longer function independently that is provided at home or an institutional setting. Respite care for 4 weeks per year is 100% covered.

Required Categories:

14. Out of pocket costs and Premium: This is the money that individuals pay to use health care services. Co-payments are not required for basic preventive services or routine screening tests.

Tier 1: Deductible. Individuals pay up to the first 3000 Swiss francs of costs each year for adults and the first 700 Swiss francs per child.

Co-insurance and co-payments. Individuals pay 15% of costs and 50 Swiss francs per hospital day and ER visit and 10 Swiss francs per doctor visit until they have paid 1000 more Swiss francs per adult and 500 Swiss francs per child.

Tier 2: Deductible. Individuals pay up to the first 2500 Swiss francs of costs each year for adults and the first 600 Swiss francs per child.

Co-insurance and co-payments. Individuals pay 10% of costs as well as 10 Swiss francs per hospital day until a maximum of 700 additional Swiss francs per adult and 350 Swiss francs per child.

Tier 3: Deductible. Individuals pay up to the first 2000 Swiss francs of costs each year for adults and the first 400 Swiss francs per child.

Co-insurance. Individuals pay 5% of costs until a maximum of 500 additional Swiss francs per adult and 250 Swiss francs per child.

15. Premium subsidy:

Tier 1: Subsidies are given to the lowest income persons and families. There is a sliding scale for amount based on income and family size. About 1/5 of population will receive a subsidy.

Tier 2: Subsidies are given to lowest income persons/families and lower-middle income persons and families. There is a sliding scale based on income and family size. About 1/3 of population will receive a subsidy.

Tier 3: Subsidies to low and lower-middle income persons and families. Any family that qualifies for subsidy will receive a full subsidy for children in the family.

16. Specialists:

Tier 1: Services are provided by a specific group of primary care doctors who deliver most of the patient care. To see a specialist, a primary care provider must make a referral. Choice of doctors and hospitals is limited. Access to the primary care doctor is much easier than access to the specialist.

Tier 2: Services are provided by a network of primary care providers and specialists. Choice of primary and specialty doctors and hospitals is greater than Tier 1 but limited within the network. Out of network specialist visit requires referral from primary doctor.

Tier 3: There is wide choice of doctors and hospitals in the community. Referral from primary doctor is not needed to see a specialist.

17. Time with the doctor:

Tier 1: You get to see your doctor less often and have less time to spend with your doctor.

Tier 2: You get to see your doctor more often and have more time to spend with your doctor.
